# The Role of Finding Out in Type 2 Diabetes Management among West-African Immigrants Living in the UK

**DOI:** 10.3390/ijerph18116037

**Published:** 2021-06-04

**Authors:** Folashade Alloh, Ann Hemingway, Angela Turner-Wilson

**Affiliations:** 1 Department of Allied and Public Health Professions, School of Health, Sport and Bioscience, University of East London, London E15 4LZ, UK; 2 Department of Public Health and Human Sciences, Faculty of Health and Social Sciences, Bournemouth University, Bournemouth BH1 3LH, UK; aheming@bournemouth.ac.uk (A.H.); aturnerwilson@bournemouth.ac.uk (A.T.-W.)

**Keywords:** immigrants, type 2 diabetes, qualitative, West-Africa, diagnosis, management

## Abstract

Type 2 diabetes (T2DM) prevalence is three times higher among West African Immigrants compared to the general population in the UK. The challenges of managing T2DM among this group have resulted in complications. Reports have highlighted the impact of migration on the health of the immigrant population, and this has contributed to the need to understand the influence of living in West Africa, and getting diagnosed with T2DM, in the management of their condition in the UK. Using a qualitative constructivist grounded theory approach, thirty-four West African immigrants living in the UK were recruited for this study. All participants were interviewed using Semi-structured interviews. After coding transcripts, concepts emerged including noticing symptoms, delayed diagnosis, affordability of health services, beliefs about health, feelings at diagnosis, and emotions experienced at diagnosis all contribute to *finding out* about diagnosis T2DM. These factors were linked to living in West Africa, among participants, and played significant roles in managing T2DM in the UK. These concepts were discussed under *finding out* as the overarching concept. Findings from this study highlight important aspects of T2DM diagnosis and how lived experiences, of living in West Africa and the UK, contribute to managing T2DM among West African immigrants. The findings of this study can be valuable for healthcare services supporting West African immigrants living in the UK.

## 1. Introduction

Type 2 Diabetes Mellitus (T2DM) is a public health concern globally. T2DM is a major public health challenge in the UK due to the increasing prevalence of the condition in the country. Specifically, there are more than 4 million people living with T2DM, an increase from 2% in 1999 to over 7% in 2019 [1,2]. The National Health Services (NHS) spend more than 10% of its budget on managing T2DM annually [3,4]. However, immigrant population are disproportionately affected by this condition [5]. Immigrant populations have been reported to be disproportionately affected by T2DM prevalence with more than 3 times higher risk among African immigrants, particularly among the West African population, compared to the white population [6]. In addition, West African immigrants are reported to have poorer T2DM management when compared to the general population in the country [7]. The increasing challenges of managing T2DM are impacted by delays in the diagnosis of the condition. T2DM onset has been reported to occur 4–7 years before the condition is clinically diagnosed [8]. However, immigrant populations present even later for T2DM diagnosis [9]. Delayed diagnosis contributes to the complications reported in living with T2DM, which has resulted in poorer management of T2DM reported [10]. To begin, studies have reported higher rates of T2DM complications, including kidney failure, amputations, blindness, and retinopathy among Africans in Africa because of delayed diagnosis [11,12]. These challenges can be associated with cultural and environmental factors that impact accessing healthcare services [13]. This study is important as African immigrants, particularly West African immigrants, are among the fastest-growing immigrant populations in the UK with over 1.3 million (17%) living in the UK [14,15].

To understand the challenges faced by West African immigrants, in managing T2DM in the UK, it is important that research explores the diagnosis process of the population. This study focused on first-generation West African immigrants who have experienced living in the Africa region.

Studies have reported on the experiences of West African immigrants in the UK [16]. However, no study has been conducted among this population to understand their experiences before migration to the UK and how these experiences impact their current T2DM management in the UK. It is important to understand this aspect of their lives, as exploring their management in isolation of their prior experience may not give a holistic picture of their experiences. In addition, understanding these individuals’ experiences in receiving T2DM diagnosis can impact the support offered in managing their condition.

Therefore, the study aims to explore West African immigrants’ knowledge and perception of being diagnosed with T2DM, and how these impact managing the T2DM condition, in the UK.

## 2. Materials and Methods

This study is part of a research project on West African immigrants living with T2DM in the UK with the purpose of understanding their experiences of living with T2DM. The current challenges experienced in living with T2DM among this population have been published [17]. However, it was important to explore aspects beyond the current management of their condition to their prior experiences that influence the management of T2DM currently in the UK. This project was approved by Bournemouth University ethical committee, UK (Approval number 13441). All participants consented to participate in this study.

### 2.1. Participants

The process of participant recruitment has been described in a previous publication [17]. Briefly, we recruited West African immigrants, living in the UK, who have been diagnosed with T2DM. Majority of individuals recruited were diagnosed with T2DM in West Africa (see Appendix A). Qualitative data was collected from participants to help understand the influence of their experiences of living in West Africa and how this might contribute to their current approach to managing T2DM in the UK. Participants were recruited through support groups and within communities (see Table 1). Of the 50 participants approached to participate in the study, 34 accepted while 16 declined. From those that declined, reasons offered include confidentiality concerns, workload, and preference of a quicker research approach, such as a questionnaire.

### 2.2. Data Collection

#### Interview

The first author (FA) conducted data collection using semi-structured interviews. The questions asked focused on the process of diagnosis, these include: Where were you diagnosed, how was the experience of being diagnosed with T2DM, what are the changes that has occurred as of the diagnosis, factors impact on your diagnosis experience, do think your environment impacted on the diagnosis and why? In addition, discussions focused on the process of diagnosis with T2DM and the impact of living in West Africa on T2DM management in the UK. Prompt questions were used to expand on participants’ responses and clarification where required. Each interview session lasted an average of one hour. All interviews were conducted in English, because participants could communicate in the language. All interviews were audio-recorded, with participants’ consent, and transcribed for analysis. The other authors (AH and ATW) reviewed interview transcripts.

### 2.3. Data Analysis

The interview transcripts were analysed using Constructive Grounded Theory (CGT) broad concept abductive approach [18]. Computer software (Nvivo 11 by QSR international) was used to support the analysis of collected data. Initially, open coding was carried out according to Charmaz [19], using this approach; each transcript was labelled, line by line, with codes that support the narrations of participants. All transcripts were open coded and focus coded to derive substantive codes. Throughout the analysis, constant comparison was done to incidents within each transcript and between transcripts. Emerging concepts were related to each other as analysis progressed to give further insight into the experiences of participants. Using the bottom-up approach, codes were identified, and they formed concepts, while concepts were aggregated to form the category that explained the experiences of participants. Memos were written throughout the process of analysis to help expand on the conceptual understanding of the findings from this study [20]. Each transcript was read multiple times to understand the underlying meaning in participants narrations. This was followed by story written from the understanding of each narration.

To ensure credibility in the analysis process, FA carried out coding and category development. AH and ATW reviewed the analysis separately, and all authors discussed and reached a consensus where disagreements occurred. In addition, the findings from this study were presented at the support groups in line with the triangulation method [21]. Fictitious names were used to ensure confidentiality of participants’ identity. To present the findings from participants, data excerpts were used to support the findings. The fictitious name, age, gender, and place of diagnosis of each participant follow supporting quotes to provide a context of each participant.

## 3. Results

Theoretical Category: *Finding Out.*

This theoretical category was identified as ‘*finding out’* which represents the process of being diagnosed with T2DM. These factors influence the management regime of each participant and future management that, in turn, influenced the ways blood glucose is controlled. Participants described how they found out about their T2DM, and the impact of *finding out* on recommended T2DM management regimes by healthcare practitioners, as a category that emerged from this study. Several concepts further explained factors that contribute to the management of T2DM among West African immigrants in the UK.

### 3.1. Noticing Symptoms

One of the key concepts, found in this study, is noticing symptoms. This concept outlines the stage at which participants began experiencing health challenges and the realisation that there is a need to seek support with their health. Participants reported symptoms including urinating frequently, fatigue, feeling thirst, weight loss, as well as mouth and body ulcers. The noticing of symptoms gives specific reasons participants felt something was not right about their health and needed to seek medical attention. This was mainly reported among participants diagnosed in the UK. All but four participants expressed noticing at least one form of symptom, which made them seek medical attention from healthcare facilities prior to *finding out.*

*“I found out, … the way it started I was feeling weak, dizzy and dry mouth, dry lip when I shouldn’t. I was filling my mouth with everything I could but it was not helping”*

(Bamba, 65 years, female diagnosed in the UK)

And:

*“I noticed a symptom of diabetes which is the frequency of urination and on a consultation of my doctors, I asked that I was having to drink a lot and a lot urinate frequently and they agreed to start the process of diagnosis of diabetes”*

(Bobaro, 57 years, male diagnosed in the UK)

However, there were reports of delay in diagnosis among participants. Responses such as “getting worse” or “family insistence” made participants seek medical explanations, which led to a diagnosis with T2DM. The participants discussed that what prompted them to notice something was not right about their health was the frequency at which the symptoms occurred.

It became apparent that people in this study not only noticed symptoms but also experienced an increase in symptoms occurrence that propelled them need to seek medical attention. However, most tried local medicines and self-treatment before accessing medical healthcare services as a last resort. In taking this further, the frequency of symptoms was perceived as more severe when they interfered with daily life of participants. The analysis found that the severity of symptoms, which interfere with daily activities of participants, made them seek medical attention.

As highlighted, finding out about T2DM among participants was influenced by the environment (location) of diagnosis. T2DM diagnosis in West Africa differs significantly from diagnosis in the UK. For participants that were diagnosed in West Africa, delayed diagnosis affected their management of T2DM.

Most participants, particularly those diagnosed in West Africa, continuously tried home remedies to manage the symptoms before seeking medical help. The unawareness and the environment influenced the length of delay before seeking medical support and diagnosis.

*“But you know our culture, it was thought to be something else my sexual life was affected, tried with my wife; it was the same thing so I concluded that there was something wrong, it began to sink in that I had diabetes. Because of that side effect it sank in that I had diabetes, I tried all African herbs that I could think of you know”*

(Konge, 45 years, male diagnosed in West Africa)

### 3.2. Delayed Diagnosis

Being unaware of the importance of early diagnosis among most of the participants contributed to the delayed diagnosis reported in this study. Participants discussed how lack of awareness contributed to delay in seeking medical attention when symptoms were noticed in West Africa.

*“So my mum had diabetes, I used to have palpitations, but I didn’t know what they were each time I get angry or upset, it’s palpitations. So I think it also worked with my blood sugar, yes but I didn’t know I had blood sugar, I didn’t know”*

Kenfa 52 years, male diagnosed in West Africa

And:

*“That is something that we never took as it would affect us because …. Ignorance is the biggest killer”*

(Dorima 37 years, male diagnosed in West Africa)

As the analysis progressed, the location (West Africa or the UK) of diagnosis influenced how participants found out about their T2DM status. This highlights the need to go beyond individual factor in the *finding out* concept. The environmental influences include social norms of using alternative medicines, limited access to healthcare services, lack of medical services availability, and health beliefs in West Africa were discussed as contributing factors to delay seeking medical attention for T2DM diagnosis. Participants living in West Africa, at the time of diagnosis, discussed how they delayed seeking medical attention. This was attributed to several factors in the environment.

*“There is the issue of misdiagnosis or unknown condition in Africa, making it difficult to properly treat. Lack of knowledge about diabetes also contributes to the issue. I would say there is better management of diabetes in the UK than Africa”*

(Mubisa 69 years, female diagnosed in West Africa)

And:

*“I noticed my symptom as went to the hospital, they found nothing and sent me back home in 1987, it was when I came to the UK in 1999 that I was diagnosed as having diabetes”*

(Junde 82 years, male diagnosed in the UK)

Participants discussed how the limited availability of medical facilities in the environment (West Africa) affected accessing medical advice. It was mentioned that challenges of inaccessible medical facilities contributed to the difficulty of seeking medical attention after noticed symptoms.

*“Well, I was in my country (Nigeria) when I was diagnosed with diabetes. I was feeling very tired and thirsty initially I thought it was the stress that I was going through so I did not do much about it. It later became apparent that something was not right with my health. This was when my wife insisted I go to the hospital. I went to a private hospital because it is easier and faster to see the doctor there. It was there that I was asked to run some test and was then diagnosed with diabetes”*

(Orisa 52 years, male diagnosed in West Africa)

Following further analysis, it emerged that the ease of accessing medical attention in the UK contributed to early diagnosis among participants diagnosed in the UK. The easy accessibility of medical facilities by the population allowed most participants, diagnosed in the UK, to seek medical attention almost immediately after symptoms were noticed. For example, four participants were diagnosed with T2DM from routine checks before even noticing symptoms.

*“One thing about me … I am very concerned about my health, so I go on to check a lot … do you understand? So, I just go there to do the general test, my blood and all general. So, I was like okay do this for me … as my mum got it so let me know if am close or so. So after doing that for like … after some time they sent me a letter, that I have to go and do a further test or something like that and then that I had to go to a group and start going for lectures so I can prevent”*

(Dee 34 years, male diagnosed in the UK)

### 3.3. Affordability of Healthcare Services

As analysis progressed, the affordability of healthcare services become important to the *finding out* concept. Among participants who were diagnosed with T2DM in West Africa, the cost of accessing health services in Africa was part of the reasons for the delay in presenting health conditions to medical professionals. They discussed how paying their own medical costs impacted their access to medical facilities as the last resort, which resulted in worse symptoms before diagnosis. However, participants diagnosed in the UK did not mention cost as a factor. This might be because of the non-payment at the point of accessing healthcare service in the UK, so participants presented earlier for medical attention, when symptoms were noticed, than those diagnosed in West Africa.

*“I felt I might need to see a doctor for these symptoms but I had to get some money to register at the private hospital. So am not sure exactly how long but it took a while to go anyway, with my wife’s insisting I go when she got worried about my symptoms. Going to the general hospital is not a pleasant experience was why I waited to go to private”*

(Kenfa 52 years, male diagnosed in West Africa)

And:

*“Well … we don’t really have the opportunity to check our sugar level on routine checks … it cost money to do that so we only check when we notice concerns”*

(Kelora 57 years, male diagnosed in West Africa)

### 3.4. Beliefs about Health

Prejudgement about health was influential in seeking medical attention for symptoms noticed in T2DM diagnosis. Environmental beliefs about health contribute to when and how participants seek medical support for symptoms noticed. In most cases, participants diagnosed in West Africa expressed beliefs that other factors, such as witchcraft or religious belief about the cause of symptoms, which differs from allopathic understanding.

*“But you know our culture, it was thought to be something else, someone has bewitched me so I concluded that something was wrong”*

(Orisa 52 years, male diagnosed in West Africa)

And:

*“For me, I have faith in God, how our health turns out does not depend on our actions, God has destined what will happen irrespective. This has been my focus to make sure I live life the best way God want me to”*

(Kinjile 72 years, male diagnosed in West Africa)

And:

*“I believe I need to seek medical attention when I noticed the symptoms for my condition, however, I wanted to try other readily available self-medication. Medical service back home is not the easiest especially in government hospitals, so I tried to help myself on my own first”*

(Vero 55 years, female diagnosed in West Africa)

### 3.5. Feelings at Diagnosis

Going further into the *finding out* brought the analysis to participants’ emotions about getting diagnosed. Being diagnosed was described as a turning point in their life and marked the beginning of living with T2DM. It was explained that being diagnosed is a significant, and unforgettable, event in their life. This led to different feelings after diagnosis that is explained below.

*“Well … being diagnosed with diabetes was one of the worst news I have received ever … it was disappointing and really worrisome. I was so shocked especially because of the limited knowledge we have in Africa concerning the disease”*

(Kelora 57 years, male diagnosed in West Africa)

And:

*“It was just not something I suspected even when I was not feeling well, diabetes was beyond strange for me until I was diagnosed …. I was scared for my future, what will happen to me”.*

(Wilo 63 years, female diagnosed in West Africa)

Most participants in this study discussed the feelings that they experienced after being diagnosed with T2DM. The shock was discussed as the first emotion experienced at the discovery of having T2DM because of the unexpected event of being diagnosed. Although two participants talked about suspecting T2DM when some symptoms were noticed, it still came as an enormous shock to confirm a positive T2DM status.

*“However, the sudden shock passed and I started to think of how I can logically live with the disease and still be as normal as possible. Anyway, it has been by God’s grace so far since then we have been able to give thanks to God on the journey”*

(Willo 63 years, female diagnosed in West Africa)

### 3.6. Experienced Emotions

After the shock of being diagnosed with T2DM, panic at the realisation that they will live with T2DM for the rest of their life is another emotion that was well discussed among most participants. The panic feeling affects how they manage T2DM because of the prognosis of the condition.

One participant discussed how the initial panic of *finding out* about her T2DM made her buy all meals labelled as T2DM food. She also talked about not enjoying these foods, but her panic state made her consider so many other options in the effort to manage her T2DM. The feeling described was as though it was a death sentence when informed of T2DM diagnosis, and this made her feel vulnerable about her health.

*“I have always been health conscious, … the very first time they said I am diabetic …. In any situation, you panic, in my panic, I was going around looking for diabetic meals and I actually picked a few things that said it’s a diabetic meal. I prepared it and it was horrible, I couldn’t take it”*

(Yaranto 67 years, female diagnosed in West Africa)

Panic because of the extra burden of being diagnosed with T2DM was another feeling that participants had after diagnosis. Most participants felt that being diagnosed with T2DM has placed extra burden on their health. This is particularly because of the comorbidity of T2DM with other health conditions. Feeling panic is mainly towards the burden of managing T2DM.

*“You know I have arthritis and so I cannot walk properly, having diabetes scared me so much. I just feel my issues have double now”*

(Orisa 52 years, male diagnosed in West Africa)

And:

*“I have to tell you, I have heart issues that am already managing, to then be diagnosed with diabetes just makes me feel my heart will be overwhelmed with these issues, it will not be able to manage as long as it should”*

(Bobaro 57 years, male diagnosed in the UK)

Further analysis showed that uncertainty about the future mainly caused panic and fear among participants. This takes the analysis to a higher conceptualisation, which helps in understanding the emotions expressed at being diagnosed with T2DM. The decisions made in the early phase, after diagnosis, seem to affect the management of T2DM as time goes on. This highlights the importance of the initial phase in the management process.

### 3.7. Influence of Diagnosis on Management

Being diagnosed contributed to the management regime that participants follow to control their T2DM. All participants diagnosed in West Africa were placed on medication to manage their T2DM immediately after diagnosis, which is in addition to lifestyle changes such as dietary and physical activity.

*“They said yes I have got diabetes Type 2 and on that same day they put me on metformin. And I have been on metformin ever since from that day on”*

(Yaranto 67 years, female diagnosed in West Africa)

And:

*“I was diagnosed by chance as I was made to do a general test before retirement, I only noticed I had grown lean but nothing alarming before then. I was immediately placed on medications because of the late stage of my condition”*

(Yerityaya 77 years, male diagnosed in West Africa)

However, more participants diagnosed with T2DM in the UK were recommended lifestyle changes only to manage their sugar level. One participant explained he is still on lifestyle changes to manage T2DM without being placed on any medication. Similarly, another participant discussed how he lived with his T2DM for over 10 years on lifestyle management before being placed on first-line T2DM medication. All participants diagnosed in West Africa were immediately placed on T2DM controlling medication after diagnosis because of the delayed diagnosis in such an environment.

*“I was able to manage without … taking any tablet (Metformin) for 10yrs … I started taking the tablet in 2015 less than 4 yrs. ago so and ummm …. it has been relatively good control of diabetes”*

(Bobaro 64 years, male diagnosed in the UK)

**Figure 1:**
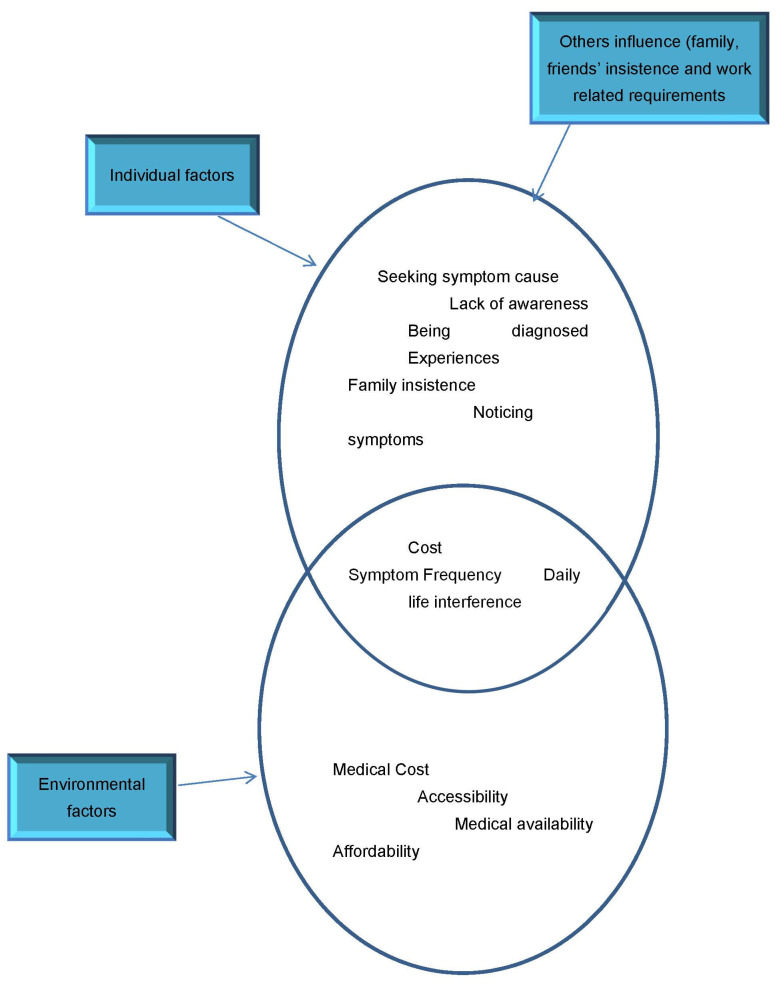
Contributing factors to *Finding Out* of T2DM.

The analysis showed that findings emerged into individual and environmental (external) factors. The upper circle (Figure 1) shows codes relating to individual factors that are within control of individuals, in seeking medical attention to symptoms, while the lower one shows the environmental (external) influence that contributes to the finding out process. The influence of family and friends, that encouraged and supported the individuals noticing symptoms to seek medical attention, can be seen as within individual control. The lower environmental (external) circle shows factors that are beyond participants’ control in seeking medical attention. This is more pronounced in West Africa, where the availability of health, accessibility, and affordability can be challenging to achieve. Their environment was actively involved in finding out. The overlap between the two circles shows how both individual factors and environmental factors interact to influence the finding out of T2DM. This was apparent in the stories of participants that found out about their T2DM status in relation to the location of diagnosis.

**Figure 2:**
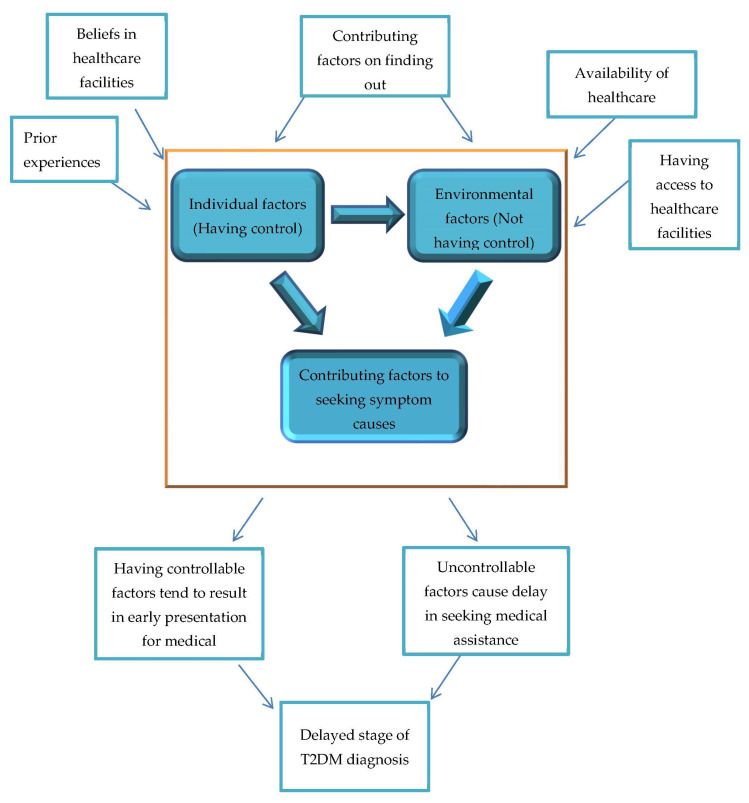
Controllable and Uncontrollable factors by individuals in finding out.

In conclusion, different factors affect the finding out concept and how these interact, to impact the T2DM management process, among West African immigrants in the UK is an important exploration. In terms of the interaction between these contributing factors, individual factors are less pronounced and influential among people diagnosed with T2DM in West Africa. Environmental barriers to finding out were more influential in the diagnosis of participants from this population. Individual factors were less impactful in being diagnosed in West Africa (see Figure 2). This means that individuals’ choice on finding out about their T2DM was restricted due to the unavailability and inaccessibility of health care facilities in this environment.

On the other hand, people diagnosed in the UK emphasize the influence of individual factors on finding out about T2DM. In this environment, there is better availability of medical services with easy access to these services. In this study, participants diagnosed in the UK reported that getting diagnosed is more dependent on individual factors such as busy schedule or not placing importance on healthcare services. These barriers are factors that are mainly influenced by self-efforts to seek the cause of their symptoms.

## 4. Discussion

This study aimed to expand West African immigrants’ experiences, and perception, of being diagnosed with T2DM and how this impacts managing the T2DM condition in the UK. The impact of the *finding out* concept showed factors that directly, and indirectly, contribute to the management of T2DM. In understanding the management of T2DM among West African immigrants in the UK, it is apparent that the experiences of living in West Africa have impacted the management approach among West African immigrants living in the UK. As stated by Romano [22] experiences are part of life that shapes us, and we will never be the same after undergoing such events. They unconsciously contribute to our perceptions of the future, beliefs and actions, irrespective of our environment. It can be inferred that West African immigrant’s lifestyle practices in their management of T2DM are outcomes of their lived experiences in West Africa prior to migration to the UK. It is essential to improve the management outcome of West African immigrants because of poor management reported.

The theoretical concept ‘*finding out’* highlights different aspects of the journey of West African immigrants in the diagnosis of T2DM, which relates to lived experiences. This category explained the interactions between participants and the healthcare system prior to diagnosis and the influences on the management of T2DM in the UK. This further highlights how participants situate themselves as patients who are shaped by their lived experiences, such as accessing healthcare services before migration to the UK. In the narrations of participants in this study, all participants draw on their experiences of accessing healthcare services in West Africa, which influences their management of T2DM in the UK. These experiences were majorly unpleasant and significant in *finding out* about their T2DM condition, as it resulted in delayed diagnosis. This is particularly pronounced among people that were diagnosed in West Africa. Alzubaidi et al. [23] reported that negative experiences, in accessing healthcare services, can be barriers to accessing healthcare services in the management of T2DM. This may explain the delay in diagnosis that most participants discussed in their narration.

All participants diagnosed in West Africa were diagnosed after noticing symptoms, highlighting the delay before getting tested for T2DM. Similar findings have been reported in the literature in terms of delayed diagnosis [24]. The delayed diagnosis has also been reported among immigrant populations living with T2DM [10,25]. In addition, the delay in diagnosis, among participants in this study, has also been reported in other research among African immigrants [4,26]. Although T2DM onset can begin 10 years before the diagnosis of the condition [8], this may be longer among West Africans where limited access to T2DM testing and diagnosis is noted.

Delay in diagnosis has been implicated for higher risks of complication, which can cause mortality [8,10,27]. For example, because of the delayed diagnosis among Africans, survey research reported that 21–25% of patients already have developed retinopathy complications of untreated T2DM [28]. Nephropathy prevalence was reported to be between 32–57% with a mean duration of 5–10 years. In addition, lower extremity amputations can be up to 7% of T2DM patients and about 12% of T2DM patients hospitalised have foot ulceration, as reported in the study [28]. However, the study did not further investigate the cause of the delayed diagnosis as a common occurrence among people in these communities. The delay in diagnosis is an event that, in most cases, leads to difficulty in meeting dietary and lifestyle recommendations in managing T2DM among this population.

The environment, in which individuals live, influences the *finding out* about T2DM and the management of the condition. In this aspect, the limited availability of healthcare services in the West African region has affected the *finding out* process. The impact of this is more significant in West Africa, where access to healthcare services is limited [29]. Some issues experienced by West African immigrants in accessing healthcare services in West Africa can influence their management of T2DM, due to the limited resources allocated to healthcare services.

Negative experiences and beliefs about healthcare services made seeking medical attention for symptoms noticed the last resort. In the findings of this study, participants express how they try other sources of treating their symptoms before going to a healthcare facility as a later resort. Similarly, Alzubaidi et al. [30] reported the delay of healthcare services among Arabic speaking immigrants, by accessing as a last resort, after the use of alternative treatments. This shows beliefs in other, alternative options to treat T2DM prior to accessing healthcare services.

In addition, there was a lack of trust, in terms of beliefs about efficiency of healthcare services, in West African environment. This may explain the lack of awareness about seeking medical explanation of noticed symptoms referenced by most of the participants in this study. Having information about healthcare availability can be influential in managing T2DM [31]. There is a lack of routine testing services in West African areas, which may be a barrier to early diagnosis of West African immigrants prior to noticing symptoms [32]. In addition, there is the issue of out-of-pocket payment, in West Africa, for patients to get diagnosed and treated for T2DM. This influenced the lack of belief that some participants express in healthcare services, which, in turn, contributes to the late diagnosis.

Similarly, Social Determinants of Health (SDH) have been implicated in the poorer management of non-communicable diseases in Africa [33,34]. The findings from this study agree with the impact of SDH among West African immigrants. This is highlighted in the explanation of environmental influence in the delayed diagnosis and poorer management of T2DM among participants prior to migration to the UK. In terms of the influence of *finding out* the concept in the management of T2DM in the UK, participants continued to refer to their experiences of lifestyle habits during, and prior to, the diagnosis of T2DM. This highlights the need to improve the management of T2DM while understanding the impact of lived experiences among the West African population.

## 5. Conclusions

The understanding of *finding out,* among participants, presents the impact of getting diagnosed with T2DM in West Africa and the UK. The influence of living in West Africa contributes to the *finding out*, getting diagnosed with T2DM, and managing the condition in the UK. Findings from this study, on the T2DM diagnosis process among West African immigrants, can be valuable to health professionals supporting this group with managing T2DM in the UK. This study highlights the importance of lived experiences of West African immigrants in the management of T2DM. Managing T2DM goes beyond current presentations in the UK, but should include understanding the lived experiences of immigrants before migration.

## Figures and Tables

**Table 1:** Participants characteristics.

Demograph Number		Percentage (%)
Gender		
Female	19	55.8
Male	15	44.1
Age Range		
30–40	4	11.7
40–50	10	29.4
Above 50	20	58.8
Employment		
Employed	20	58.8
Retired/Out of Job	14	41.2
Education		
Tertiary Level Education	21	61.8
Secondary Education	9	26.5
Primary	4	11.8
Place of diagnosis		
West-Africa	21	61.8
United Kingdom	13	38.2
Nationality		
Nigerian	22	64.7
Ghanaian	8	23.5
Gambian	4	11.8

## Data Availability

Presented data from this study are available upon considerable request from corresponding author.

## Appendix A. Participants’ Demographic Information

**Participant (Fictitious Name)**	**Age**	**Gender**	**Years of Diagnosis**	**Place of Diagnosis**	**Years of Migration**	**Employed**
Bobaro	57	M	14	UK	22	No
Ken	58	M	19	West Africa	17	Yes
Orisa	52	M	13	West Africa	10	No
Bamba	65	F	15	UK	40	No
Junde	82	M	21	UK	34	No
Lolily	63	F	14	UK	46	No
Konge	45	M	15	West Africa	10	Yes
Dee	34	M	02	UK	05	Yes
Bolu	49	F	11	West Africa	10	Yes
Wemi	56	F	05	UK	07	Yes
Mam	64	F	07	UK	20	No
Yeriyaya	77	M	16	West Africa	12	No
Kinjile	72	M	18	West Africa	15	No
Gambo	48	M	12	West Africa	07	Yes
Kantita	40	F	04	UK	08	Yes
Bolula	48	F	12	West Africa	10	Yes
Yaranto	67	F	20	West Africa	10	No
Mange	57	M	10	UK	12	Yes
Kilanla	65	F	09	West Africa	08	Yes
Vero	55	F	10	West Africa	06	Yes
Nimbabo	49	M	10	UK	15	Yes
Zurisa	69	M	22	UK	25	No
Balabi	78	M	25	West Africa	19	No
Josera	48	M	07	West Africa	05	Yes
Sisato	39	M	05	West Africa	04	Yes
Sambila	75	M	14	UK	16	No
Hena	50	F	06	UK	10	Yes
Wilo	63	F	12	West Africa	11	Yes
Kenfa	52	M	11	West Africa	10	Yes
Dorima	37	M	04	West Africa	02	Yes
Malisaya	44	M	08	West Africa	04	Yes
Mubisa	69	F	12	West Africa	07	Yes
Kelora	57	M	06	West Africa	02	Yes
Egbede	64	M	11	West Africa	07	No
